# Acoustic respiration rate and pulse oximetry-derived respiration rate: a clinical comparison study

**DOI:** 10.1007/s10877-018-0222-4

**Published:** 2018-11-26

**Authors:** Michal E. Eisenberg, Dalia Givony, Raz Levin

**Affiliations:** 1 Department of Rehabilitation, Herzog Hospital, Jerusalem, Israel; 2 Medtronic, 7 HaMarpe st, 97774 Jerusalem, Israel

**Keywords:** Respiration rate, Pulse oximetry-derived respiratory rate (RRoxi) acoustic respiration rate (RR_a_), Capnography

## Abstract

Respiration rate (RR) is a critical vital sign that provides early detection of respiratory compromise. The acoustic technique of measuring continuous respiration rate (RR_a_) interprets the large airway sound envelope to calculate respiratory rate while pulse oximetry-derived respiratory rate (RR_oxi_) interprets modulations of the photoplethsymograph in response to hemodynamic changes during the respiratory cycle. The aim of this study was to compare the performance of these technologies to each other and to a capnography-based reference device. Subjects were asked to decrease their RR from 14 to 4 breaths per minute (BPM) and then increase RR from 14 to 24 BPM. The effects of physiological noise, ambient noise, and head movement and shallow breathing on device performance were also evaluated. The test devices were: (1) RR_a_, Radical-7 (Masimo Corporation), (2) RR_oxi_, Nellcor™ Bedside Respiratory Patient Monitoring System (Medtronic), and (3) reference device, Capnostream20p™ (Medtronic). All devices were configured with their default settings. Twenty-nine healthy adult subjects were included in the study. During abrupt changes in breathing, overall RR_oxi_ was accurate for longer periods of time than RR_a_; specifically, RR_oxi_ was more accurate during low and normal RR, but not during high RR. RR_oxi_ also displayed a value for significantly longer time periods than RR_a_ when the subjects produced physiological sounds and moved their heads, but not during shallow breathing or ambient noise. RR_oxi_ may be more accurate than RR_a_ during development of bradypnea. Also, RR_oxi_ may display a more reliable RR value during routine patient activities.

**Electronic supplementary material:**

The online version of this article (10.1007/s10877-018-0222-4) contains supplementary material, which is available to authorized users.

## Introduction

Respiration rate (RR) is a critical vital sign that provides early detection of patient distress and respiratory compromise, a state in which there is a high likelihood of deterioration into respiratory insufficiency, respiratory failure or death. However, during respiratory compromise, specific interventions (e.g., enhanced monitoring, therapies) might prevent or mitigate this deterioration [1, 2]. Current clinical practices for respiratory rate monitoring lag behind monitoring standards for automatically recorded vital signs such as heart rate, blood pressure, and peripheral oxygen saturation. Respiratory rate remains the least well-documented vital sign, potentially because of lack of clinical staff time, knowledge or equipment constraints [1, 3, 4, 5]. In general hospital wards RR is typically manually registered only once every shift, i.e., at best once every 8–10 h. An unnoticed period of abnormal RRs that occurs between these routine clinical assessments could lead to detrimental outcomes, such as unscheduled intensive care unit (ICU) admission and in-hospital mortality [6].

Increased RR (i.e. hyperventilation) may reflect microcirculatory failure induced by underlying medical conditions as sepsis, pulmonary embolism and congestive heart failure [7]. The microcirculatory failure causes a progressive decline in the efficiency of gas exchange in the lungs [8]. The increased minute ventilation that occurs in this state causes a greater volume of oxygen to be ventilated into the lungs, while the SpO2 levels remain unchanged, thus masking the respiratory distress of the patient. By the time the SpO2 level is 90% or below, the efficacy of treatment is reduced and the risk for hospital morbidity and mortality increases [7].

On the other hand, respiratory depression and respiratory insufficiency are among the most common precipitating causes of in-hospital resuscitation or cardiac arrest events [9–11]. Postoperative patients receiving opioid medications are particularly susceptible to in-hospital cardiopulmonary and/or respiratory arrest [12] and unrecognized opioid-induced respiratory depression remains a significant cause of in-hospital adverse events [13–16]. In postoperative patients receiving opioid medications, clinician observation, pulse oximetry, and capnography are often used individually or in combination to monitor ventilation [17–19]. Along these lines, the American Society of Anesthesiologists amended its Standards for Basic Anesthetic Monitoring to include mandatory exhaled end-tidal carbon dioxide (PetCO_2_) monitoring (i.e., capnography) during both moderate and deep sedation [20].

While capnography is often considered the “gold standard” for respiratory rate monitoring [21–25], its measurement accuracy can be adversely impacted by mouth breathing. The use of an oral–nasal cannula, which is also intended for use in the general care floor, addresses the mouth breathing effect on capnography accuracy. High flow (> 5 L/min) supplemental oxygen that is delivered through the cannula [26–28] may also affect accuracy. In addition, capnography requires the patient to wear a nasal cannula which may be poorly tolerated by some patients (as are many wearable patient monitoring technologies). Recent advances in respiratory rate monitoring technology may provide clinicians with other tools that may be better tolerated by patients who are not able to wear an oral nasal cannula, and thereby may potentially increase the use of RR monitors and reduce the incidence of adverse events related to undetected respiratory depression. The acoustic respiration rate (RR_a_) technique monitors inhalation and exhalation sounds, using an adhesive sensor with an integrated acoustic transducer that is applied to the patient’s neck. The patient’s RR is calculated based on this information. The sensor is applied in addition to the finger sensor used to measure oxygen saturation (SpO2) and pulse rate [27, 29]. Pulse oximetry-derived respiratory rate (RR_oxi_) uses a pulse oximeter signal or photoplethysmogram variations to measure pulse rate and respiratory rate via a single dedicated finger pulse oximetry sensor [30, 31]. To our knowledge, the accuracy of the RR_a_ and RR_oxi_ technologies have not been directly compared.

Thus, the primary objective of this study was to evaluate the differences in accuracy between RR_oxi_ and RR_a_ as compared to a capnography-based reference device in different conditions that mimic respiratory compromise patterns. The second objective of the study is to compare the effects of normal physiological activities and environmental phenomena on the device performance. To support these objectives, we evaluated the effects of changes in RR and depth along with the effects of physiological and environmental noises and subject head movements on RR_oxi_ and RR_a_ accuracy.

## Materials and methods

### Study population

The study population included male and female healthy subjects, not suffering from current respiratory disease; age 18–40 years, with no history of or current chronic obstructive pulmonary disease (COPD), heart disease or significant cardiac arrhythmias. Subjects were excluded if they had any breathing difficulties during the study, if they were unable or unwilling to follow the study protocol, and if they had any contact allergy to the RR_a_ adhesive neck sensor. The study protocol and informed consent form (ICF) were reviewed and approved by the Herzog Hospital Ethics Committee. All subjects received an oral and written explanation about the study from the principal investigator and signed the ICF. The study was performed in accordance with the Helsinki Declaration and good clinical practice.

### Study design and measurements

The study was a prospective observational study. The evaluated devices were the Radical-7+RAS-125 Version C (Masimo Corporation, Irvine, CA) and the Nellcor™ Bedside Respiratory Patient Monitoring System, PM1000N, with Nellcor Adult Respiratory Sensor (Medtronic, Minneapolis, MN, USA). The Capnostream™20p, with Smart CapnoLine Plus (Medtronic, Minneapolis, MN, USA) was used as a reference device. All devices were configured with the factory default settings. The Radical-7 device was set to *Adult* mode with *Adaptive Probe-Off Detection (APOD)* sensitivity mode, which is the recommended mode for care areas where patients are not visually monitored continuously (such as general care floor) or where there is a high probability of the sensor becoming detached. The length of time over which the system calculates the average of all data points was *Slow*. The Nellcor™ Bedside Respiratory Patient Monitoring System was set to *Adult* mode with *Normal* response time.

### Study procedures

Subjects were tested in the supine position on a bed. Each subject wore the adhesive RAS-125C sensor on the left side of the neck and a finger clip on the left middle finger to measure RR_a_, for the Masimo Radical7 Patient Monitoring system. Subjects wore a sensor to measure RR_oxi_ on the left index finger, for the Nellcor Bedside Respiratory Patient Monitoring System, PM1000N-RR, and wore an oral/nasal Smart CapnoLine Plus, which was attached to the Capnostream™20p to measure capnography respiratory rate (RR_ref_).

During the procedures subjects were asked to breathe in different patterns that mimic different clinically relevant presentations. The subjects followed a visual metronome that guided them regarding the expected RR and each test began with a 3 min session of coached breathing using a metronome set to 14 breaths/min (BPM) in order to set up the devices and create a baseline RR. The subjects were asked to decrease their RR from 14 to 4 BPM, a condition which may appear in patients that after sedation or patients who receive opioid analgesia [7] or to increase their RR from 14 to 24 BPM, mimicking a condition which may appear in patients with hyperventilation-compensated respiratory failure, which might be an early indication for sepsis [32], congestive heart failure or pulmonary embolism [7]. Afterwards, the subjects were asked to mimic breathing patterns of patients with obstructive sleep apnea and severely delayed arousal as a result of apnea [7, 33]; to do so, subjects were asked to hold their breath for short periods of times and then to hold their breath for as long as they could. The subjects were also asked to perform shallow breathing for 2 min.

Afterwards, subjects mimicked daily activities of non-intubated patients. Subjects performed physiological noises (i.e. groaning, snoring, talking and coughing) and heard environmental noises (prerecorded medical device alarms, talking, and music with headphones). The volume of the physiological and environmental noises was measured by a decibel meter to maintain consistency between subjects. Afterwards, the subjects shook their heads in up/down and left/right directions.

All study breathing patterns and evaluated conditions are presented in Supplemental Table 1.

### Statistical analysis

For the analysis of the accuracy of the evaluated devices during monitored RR changes (accuracy error rate and absolute value errors), a Mixed Model analysis based on the differences between the two accuracy errors were used. The non-parametric related samples Wilcoxon test with α level of 0.05 was used to analyze the data other than accuracy error rates and absolute value error. Accuracy error rates were computed for each test device using the following: accuracy error rate = |RR_tested device_ − RR_ref_|/RR_ref_. Accuracy error rates were also calculated separately for the following groups of RR: (1) when the subject had bradypnea (4–8 BPM), (2) when the subject had normal breathing (9–23 BPM), and (3) when the subject had tachypnea (24–30 BPM). The absolute value error for each device was calculated using the following: absolute value error = |RR_tested device_ − RR_ref_|. In addition, the percentage of time that the test devices displayed the same values (± 2 BPM) as the reference device was determined (percentage of accuracy time). Absolute value error and percentage of accuracy time were also calculated for the three groups of RR described above [bradypnea (4–8 BPM), normal breathing (9–23 BPM), and tachypnea (24–30 BPM)]. Values are reported as mean ± SD unless otherwise noted and statistical significance was set at *p* < 0.05 for all analyses.

## Results

### Subject demographics

The study population included 29 healthy subjects (21 men and 8 women), with an age of 23.6 ± 4.4 years, a BMI of 24.6 ± 3.8, and a neck circumference of 36.0 ± 7.4 cm. Subject skin color ranged from white to olive skin tone. Five subjects reported a prior history of smoking and six subjects were current smokers (4.67 cigarettes per day on average). One subject was treated with insulin for type 1 diabetes and one subject had a history of childhood asthma.

All the devices were well tolerated by all subjects and no adverse events were reported.

### Respiratory rate accuracy during controlled breathing

Comparing the overall accuracy errors rate and absolute value errors, RR_oxi_ was more accurate than RR_a_. When examining only periods of bradypnea, RR_oxi_ indicated similar values to the reference device for a significantly longer time than RR_a_. The absolute value error and the accuracy error rate during bradypnea of RR_oxi_ was significantly lower than that of RR_a_. However, during tachypnea, RR_oxi_ indicated similar values to the reference device for shorter time than RR_a_ but no differences in accuracy error rate or absolute value error were observed. The accuracy estimation with 95% confidence intervals (CI) for proportions of misdetection of bradypnea at the default alarm setting was 0.99% (95% CI 0.17–1.82%) for RR_oxi_ and 5.41% (3.13–7.69%) for RR_a_, resulting in a significant difference between devices (*p* = 0.002).

**Fig. 1:**
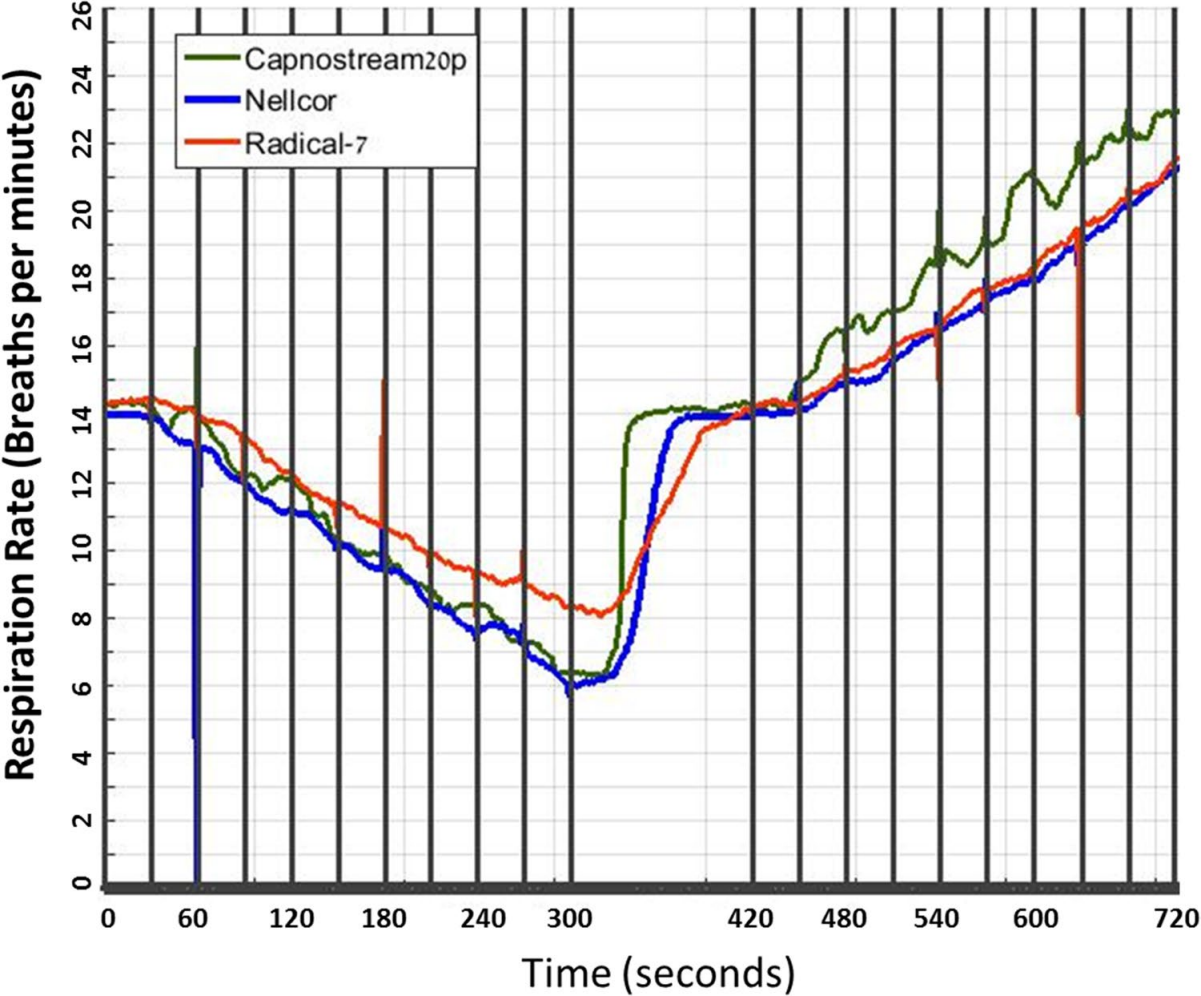
Respiratory rate during controlled breathing

The results are detailed in Table 1 and Fig. 1.

**Table 1:** Percentage of accuracy time, accuracy error rate and absolute error rate during controlled breathing

Parameter	RR_oxi_ (mean ± SD)	RR_a_ (mean ± SD)	p-value
Accuracy time			
Total (%)	81.8 ± 11.6	76.8 ± 13	0.130
Low RR (4–8 BPM) (%)	91.6 ± 21.6	65.7 ± 38.4	0.009**
Normal RR (9–23 BPM) (%)	83.1 ± 10.4	80.7 ± 11.8	0.452
High RR (24–30 BPM) (%)	26.2 ± 32.8	40.0 ± 38.4	0.019*
Accuracy error rate			
Total	0.095 ± 0.029	0.132 ± 0.059	0.006**
Low RR (4–8 BPM)	0.16 ± 0.12	0.30 ± 0.21	0.003*
Normal RR (9–23 BPM)	0.08 ± 0.02	0.1 ± 0.04	0.032*
High RR (24–30 BPM)	0.14 ± 0.05	0.13 ± 0.06	0.125
Absolute error rate			
Total	1.36 ± 0.44	1.67 ± 0.62	0.03*
Low RR (4–8 BPM)	1.11 ± 0.92	2.06 ± 1.53	0.003**
Normal RR (9–23 BPM)	1.31 ± 0.38	1.54 ± 0.56	0.087
High RR (24–30 BPM)	3.54 ± 1.31	3.20 ± 1.51	0.133
Misdetection of bradypnea (RR < 8) (%)	0.99 (0.17–1.82)	5.41 (3.13–7.69)	0.002**

*p-value < 0.05

**p-value < 0.01

### Respiratory rate accuracy during abrupt changes in breathing

Overall, throughout the session, RR_oxi_ was more accurate than RR_a_, with significant differences during bradypnea and normal breathing and non-significant differences during tachypnea. The estimation of the proportion of misdetection of bradypnea was 0.57% (95% CI 0–1.23%) for RR_oxi_ and 10.25% (7.08–13.42%) for RR_a_, resulting in a significant difference between devices (*p* < 0.001).

**Fig. 2:**
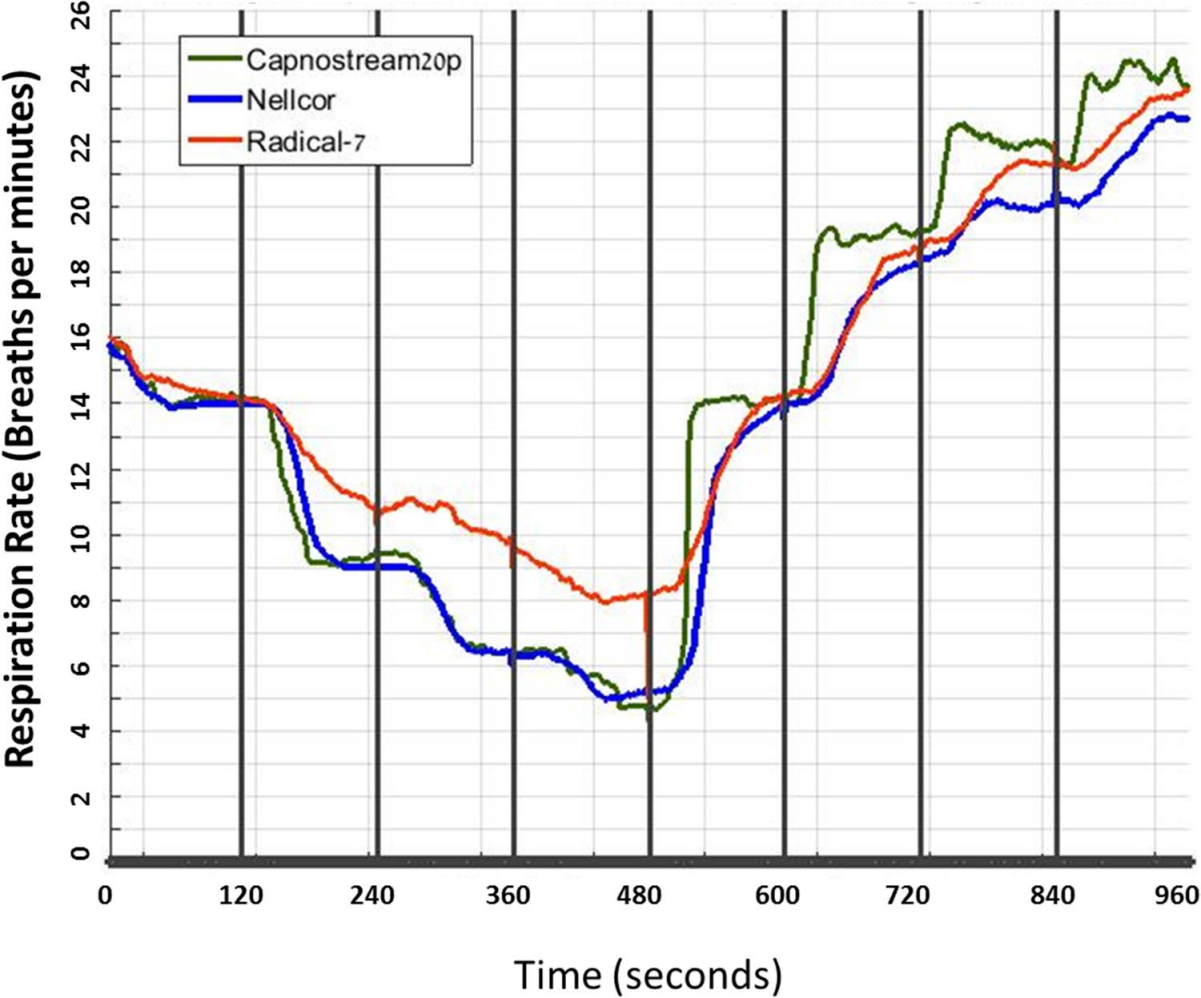
Respiratory rate during abrupt changes in breathing

The results are detailed in Table 2 and Fig. 2.

**Table 2:** Percentage of accuracy time, accuracy error rate and absolute error rate during abrupt changes in breathing

Parameter	RR_oxi_ (mean ± SD)	RR_a_ (mean ± SD)	p-value
Accuracy time			
Total (%)	77.5 ± 11.9	63.0 ± 15.7	0.028*
Low RR (4–8 BPM) (%)	88.4 ± 18.1	42.4 ± 34.4	<0.001**
Normal RR (9–23 BPM) (%)	76.2 ± 11.4	69.1 ± 15.1	< 0.001**
High RR (24–30 BPM) (%)	60.8 ± 35.5	68.4 ± 28.8	0.428
Accuracy error rate			
Total	0.118 ± 0.057	0.229 ± 0.101	< 0.001**
Low RR (4–8 BPM)	0.17 ± 0.12	0.56 ± 0.30	< 0.001**
Normal RR (9–23 BPM)	0.10 ± 0.05	0.13 ± 0.06	0.005**
High RR (24–30 BPM)	0.12 ± 0.15	0.08 ± 0.04	0.15
Absolute error rate			
Total	1.58 ± 0.98	2.19 ± 0.84	0.006**
Low RR (4–8 BPM)	1.01 ± 0.67	3.25 ± 1.83	< 0.001**
Normal RR (9–23 BPM)	1.59 ± 0.83	1.86 ± 0.76	0.125
High RR (24–30 BPM)	2.95 ± 3.61	2.05 ± 1.09	0.148
Misdetection of bradypnea (RR ≤ 8) (%)	0.57 (0–1.23)	10.25 (7.08–3.42)	< 0.001**

*p-value < 0.05

**p-value < 0.01

### Respiratory rate accuracy during altered conditions

#### Shallow breathing

During 2 min of shallow breathing (24 BPM), RR_a_ had significantly more episodes in which it did not display a value and a false alarm was triggered, as compared to RR_oxi_ (1.00 ± 0.756 vs. 0.45 ± 0.510; *p* = 0.032), but there were no significant differences in the percentage of time that the devices showed a value (RR_oxi_ = 83.8 ± 25.4%, RR_a_ = 73.6 ± 31.9%; *p* = 0.236). The total recorded time from all subjects was 3418 s. Of the 569 s that RR_oxi_ did not display a value, RR_a_ displayed the same value (± 2 BPM) as the reference device for 285 s (50.1%). Of the 996 s that RR_a_ did not display a value, RR_oxi_ displayed the same value (± 2 BPM) as the reference device for 285 s (29.5%).

#### Physiological noises

During the physiological noises session, RR_a_ had significantly more episodes in which it did not display a value and a false alarm was triggered, as compared to RR_oxi_, and the percentage of time that RR_oxi_ displayed a value was significantly longer than the percentage of time that RR_a_ presented a value. Analysis of specific physiological noises indicated that during groaning, talking and coughing, RR_oxi_ displayed a value for longer time than RR_a_ while no significant differences were seen for snoring.

The results are detailed in Table 3.

**Table 3:** Percentage of time the devices displayed a value during the evaluated physiological noise conditions

Physiological noises	RR_oxi_ (mean ± SD)	RR_a_ (mean ± SD)	p-value
Total (%)	82.4 ± 19.2	58.0 ± 14.8	< 0.001**
Groaning (%)	96.6 ± 10.4	81.6 ± 20.7	0.001**
Snoring (%)	84.2 ± 31.1	72.6 ± 32.8	0.075
Talking (%)	79.3 ± 32.7	36.5 ± 24.6	<0.001**
Coughing (%)	56.9 ± 32.3	18.9 ± 25.7	< 0.001**

*p-value < 0.05

**p-value < 0.01

#### Ambient noises

Ambient noises had no effect on the devices’ ability to display a value and both devices displayed a value for more than 98% of the time (RR_oxi_ = 98.16%, RR_a_ = 98.30%).

#### Head movements

During the head movement session, RR_a_ had significantly more episodes during which it did not display a value and a false alarm was triggered as compared to RR_oxi_ (3.00 ± 0.707 vs. 1.38 ± 1.049, respectively; *p* < 0.001). Also, the percentage of time that RR_oxi_ displayed a value was significantly longer than RR_a_ displayed a value (82.9 ± 17.3% vs. 71.1 ± 12.7%, respectively; *p* = 0.002). For head movements in lateral directions and circular movement, but not for anterior/posterior directions, RR_oxi_ displayed a value for a longer time than RR_a_ did.

The results are detailed in Table 4.

**Table 4:** Percentage of time the devices displayed a value during the evaluated head movement conditions

Head movements	RR_oxi_ (mean ± SD)	RR_a_ (mean ± SD)	p-value
Total (%)	82.9 ± 17.3	71.1 ± 12.7	0.002**
Lateral direction (%)	88.8 ± 19.4	68.7 ± 22.1	0.001**
Anterior/posterior direction (%)	82.8 ± 24.6	72.6 ± 22.9	0.082
Circular movement (%)	88.3 ± 27.7	72.7 ± 24.3	0.005**

**p-value < 0.01

#### Apnea, sensor removal, and sensor positions

The RR_oxi_ device utilized is not intended for use in identifying apnea and therefore was not part of the apnea analysis. Compared to the reference device, RR_a_ detected 26 of 63 (41.3%) breath cessation events. Of the breath cessation events, RR_a_ did not detect 4 of 14 (28.6%) events that were over 60 s and did not detect 2 of 8 (25.0%) events that were over 90 s. Apneas were detected on average 13.5 s after the reference device. For the sensor removal evaluation, the overall time to detect inactivity was 44.5 ± 3.2 s in the first session and 47.7 ± 2.4 s in the second session. Changes to the position of the RR_a_ neck sensor had no effect on the device working time (middle position = 88.6 ± 17.7%, upper third = 85.0 ± 26.1%, and lower third = 85.5 ± 19.1%).

## Discussion

In this study, the accuracy of RR_a_ and RR_oxi_ was compared during a wide variety of subject breathing rates, using capnography as a reference standard. The different breathing rates and changes in these rates were intended to simulate respiratory compromise patterns which may appear in patients with bradypnea (e.g. patients who received opioid analgesia) or in patients with tachypnea (e.g. patients with sepsis or heart failure). The impact of both physiological and ambient noise on the ability of the tested devices to display an accurate RR were also evaluated. While the overall accuracy of both methods was relatively high, the findings of the study suggest that RR_oxi_ may be more accurate than RR_a_ during the development of bradypnea. Our results also indicate that physiological noises and certain head movements are more likely to adversely impact the performance of RR_a_ as compared to RR_oxi_, suggesting that RR_a_ may be more sensitive to noise artifacts and patient movement than the pulse-oximetry-based RR_oxi_ technology.

Our study results are different from a 2013 study of 33 post-surgical patients, which found that RR_a_ compared favorably to capnography, with modest but statistically higher accuracy and precision [27]. However, in discussing the limitations of their study, the authors noted that direct observation of specific patient events that might result in inaccurate readings or data loss, such as coughing, speaking, and snoring, would help increase the understanding of the performance and limitations of the RR_a_ method [27]. Indeed, the current study findings, which was conducted in a controlled environment and conditions, demonstrated these limitations, and the performance of the RR_a_ device was seen to be reduced compared to that of the RR_oxi_ device during physiological noise challenges, and also in response to certain head movements.

A study by Kitsiripant et al. [34] compared the Nellcor™ Bedside Respiratory Patient Monitoring System PM1000N to the Radical-7®, in terms of the devices’ ability to detect apnea in volunteers. However, since the PM1000N’s intended use does not include apnea detection, the purpose of the study is not clear. In the current study, in which apnea detection with Radical-7® was compared to capnography, RR_a_ failed to detect almost 60% of breath cessation events lasting more than 30 s and 25% of events lasting more than 90 s. In our analysis, RR_oxi_ successfully detected bradypnea (< 9 BPM) over 99% of the time as opposed to a detection rate of slightly < 90% for RR_a_. This data suggest that RR_oxi_ may be better suited than RR_a_ for the continuous monitoring in patients at risk of respiratory depression, such as post-operative patients receiving opioid analgesia.

The evaluation of Nellcor™ Bedside Respiratory Patient Monitoring System PM1000N and Radical-7® during a controlled wide range of breathing patterns and rates, and in the face of physiological and ambient noise challenges, provides insight into the technical performance of these devices and may give the clinician better understanding about the functioning and accuracy of the devices in different types of patients. The study indicates that the RR_oxi_ may be preferred over RR_a_ for patients who are able to talk and cough, and for patients with abrupt changes in breathing and multiple bradypnea sessions (such as patients who have received opioids), but in patients with normal RR or tachypnea, the differences were not clinically significant.

However, controlled assessment has its limitations. First, the study employed healthy volunteers, thus the accuracy and performance of these devices in distinct patient populations, including patients predisposed to respiratory depression (i.e., patients receiving opioids and/or sedative medications and patients with significant comorbidities such as obesity or sleep apnea) remains unknown. Similarly, our analysis of non-hospitalized subjects did not and could not address whether or not the observed differences between RR_a_ and RR_oxi_ were clinically significant, although these differences were found to be statistically significant. Secondly, the study used artificial, subject-controlled changes in breathing rate that mimic bradypnea or tachypnea in patients but do not reflect the natural variations in breathing rate which would be observed in a patient population, including both sedated and non-sedated patients. Finally, the physiological noises evaluated, including groaning, snoring, and coughing, were generated by the subjects on demand rather than in response to actual physiological stimuli. Despite these limitations, our results serve to expand our understanding of the accuracy and real-world performance of RR_a_ and RR_oxi_ for the continuous monitoring of respiratory rate while addressing some of the noted limitations of prior studies.

## Conclusions

RR_oxi_ was more accurate and reliable than RR_a_ during the development of bradypnea and during routine patient activities. While the clinical implications of these observed differences are unknown, further study into the accuracy and real-time performance of these methods for continuous monitoring of respiratory rate in a true clinical setting is warranted.

## Electronic supplementary material

Below is the link to the electronic supplementary material.

Supplementary material 1 (DOCX 26 KB)
