# Supplementary material for: Acoustic respiration rate and pulse oximetry-derived respiration rate: a clinical comparison study
Source: J Clin Monit Comput. 2018 Nov 26;34(1):139–46. doi: 10.1007/s10877-018-0222-4 (PMC6946723; doi:10.1007/s10877-018-0222-4)
Supplement: Supplementary file 1 — Supplementary material 1 (DOCX 26 KB) [file 10877_2018_222_MOESM1_ESM.docx]

**Supplemental Table 1. Study Breathing Patterns and Test Conditions**

| **Controlled Breathing (Bradypnea, Normal Breathing, Tachypnea)**   - 14 BPM for 3 minutes (Baseline) - 13 BPM for 30 seconds - 12 BPM for 30 seconds - 11 BPM for 30 seconds - 10 BPM for 30 seconds - 9 BPM for 30 seconds - 8 BPM for 30 seconds - 7 BPM for 30 seconds - 6 BPM for 30 seconds - 5 BPM for 30 seconds - 4 BPM for 30 seconds - 14 BPM for 2 minutes - 15 BPM for 30 seconds - 16 BPM for 30 seconds - 17 BPM for 30 seconds - 18 BPM for 30 seconds - 19 BPM for 30 seconds - 20 BPM for 30 seconds - 21 BPM for 30 seconds - 22 BPM for 30 seconds - 23 BPM for 30 seconds - 24 BPM for 30 seconds |
| --- |
| **Shallow Breathing**   - 24 BPM for 2 minutes |
| **Abrupt Changes in Breathing**   - 14 BPM for 2 minutes (Baseline) - 9 BPM for 2 minutes - 6 BPM for 2 minutes - 4 BPM for 2 minutes - 14 BPM for 2 minutes - 19 BPM for 2 minutes - 22 BPM for 2 minutes - 24 BPM for 2 minutes |
| **Apnea**   - 6 BPM for 90 seconds (Baseline) - Hold breath for 15 seconds - 6 BPM for 90 seconds - Hold breath for 25 seconds - 6 BPM for 90 seconds - Hold breath for 35 seconds - 6 BPM 90 seconds - Hold breath for 45 seconds - 6 BPM for 90 seconds - Hold breath for as long as possible |
| **Physiological Noises^a^**   - Breathe normally (12-18 breaths/min) for 2 minutes (Baseline) - Groan for 2 minutes - Normal breathing for 30 seconds - Snore for 2 minutes - Normal breathing for 30 seconds - Talk for 2 minutes - Normal breathing for 30 seconds - Cough for 2 minutes |
| **Ambient Noises^b^**   - Breathe normally for 2 minutes (Baseline) - Hospital environmental noises introduced for 2 minutes at 65 decibels - Normal breathing for 30 seconds - Hospital environmental noises introduced for 2 minutes at 85 decibels - Normal breathing for 30 seconds - Hospital environmental noises introduced for 2 minutes at 85 decibels with peaks of 100 decibels - Normal breathing for 30 seconds - The subject heard talking for 2 minutes at 65 decibels - Normal breathing for 30 seconds - Music with headphones introduced for 2 minutes at 65 decibels. |
| **Head Movements^c^**   - Breathe at 14-16 BPM for 2 minutes (Baseline) - Move head in a lateral direction for 2 minutes (from side to side) - Normal breathing for 90 seconds - Moved head in an anterior/posterior direction for 2 minutes (up/down-shake head yes) - Normal breathing for 90 seconds - Twist head in the left/right direction for 2 minutes (shake head no) |
| **Sensor Removal**   - After 60 seconds of normal breathing, the neck sensor was removed and suspended in the air. - At the second session, after 60 seconds of normal breathing, the neck sensor was removed and placed on a pillow next to the subject. |
| **Different Positions of Masimo Sensor on Neck**   - The sensor was placed at 3 different positions on the patient’s neck (top third, middle third, and bottom third of the neck) on the side of the larynx, and the subjects breathed shallow breathing for 60 seconds and normal breathing for 60 seconds. |

### ^a^ The subjects sat during the talking and coughing sessions.

^b^ A prerecording of typical hospital noise was used to create typical environmental noise.

^c^ The subjects sat during the head movement session
